# Effect of a novel motion and simultaneous irrigation on the cyclic fatigue resistance of Hyflex EDM OneFile

**DOI:** 10.1007/s10266-024-01051-8

**Published:** 2025-01-13

**Authors:** Giusy Rita Maria La Rosa, Pietro Lombardo, Luigi Generali, Eugenio Pedullà

**Affiliations:** 1 Department of General Surgery and Medical-Surgical Specialties, University of Catania, 95123 Catania, Italy; 2 Department of Surgery, Medicine, Dentistry and Morphological Sciences With Transplant Surgery, Oncology and Regenerative Medicine Relevance (CHIMOMO), University of Modena and Reggio Emilia, 41124 Modena, Italy

**Keywords:** Dynamic cyclic fatigue, HyFlex EDM, ReFlex dynamic motion, Simultaneous irrigation

## Abstract

The aim of this study was to assess the cyclic fatigue resistance of a single-file system (i.e., Hyflex EDM OneFile), during continuous rotation and reflex dynamic motion with and without irrigation. Cyclic fatigue tests were conducted on 48 new Hyflex EDM One files using two different motions, with and without irrigation. The files were randomly assigned to four groups (*n* = 12) based on the tested motion and irrigation conditions: continuous rotation and the novel kinematics ReFlex Dynamic motion, with and without irrigation. The dynamic fatigue resistance was measured as the number of cycles to fracture (NCF) in an artificial zirconium canal with a 60° angle and a 5 mm radius of curvature at an intracanal temperature. The results were analyzed using the non-parametric Mann–Whitney test, with a significance level established at 5% (*p* < 0.05). The fractured instruments were examined with a scanning electron microscope. The instruments activated in continuous rotation, irrespective of irrigation conditions, demonstrated significantly higher NCF compared to those in ReFlex Dynamic (*p* < 0.05). Irrigation significantly impacted the cyclic fatigue resistance of Hyflex EDM in ReFlex Dynamic motion (*p* < 0.05), while it did not influence the resistance of files tested under continuous rotation (*p* > 0.05). Within the limitations of this in vitro study, HyFlex EDM One files tested under continuous rotation showed greater resistance to cyclic fatigue compared to those tested with ReFlex Dynamic motion, regardless of irrigation. Continuous hypochlorite irrigation enhanced the cyclic fatigue resistance only of the files tested in ReFlex Dynamic motion.

## Introduction

Nickel–titanium (NiTi) rotary files have profoundly transformed the concept of root canal preparation. Despite their numerous advantages, these instruments are susceptible to fracture, especially when used in curved canals [1]. Fractures can result from either torsional failure or cyclic fatigue, with cyclic fatigue being the predominant cause of unexpected breakages [2]. Cyclic fatigue occurs due to the repetitive exposure of rotary instruments to alternating tensile and compressive stresses while in motion within curved canals [3]. The breakage of rotary files is influenced by various factors [4], including root canal geometry [2], instrument properties, and kinematics [5].

In an effort to simplify instrumentation procedures and minimize mechanical stress, single-file systems have been introduced. HyFlex EDM single files (Coltene/Whaledent, Altstätten, Switzerland) are produced using electrical discharge machining (EDM), enhancing their resistance to cyclic fatigue [6].

Also, kinematics is able to modify the cyclic fatigue behavior of NiTi files. Reciprocating motion has been reported to extend the lifespan of NiTi rotary files compared to continuous rotation [7]. The recently introduced hybrid kinematics, particularly, result in reduced file breakage and improved file progression within the canals [8]. The ReFlex Dynamic, performed by the EndoPilot^®^ endodontic handpiece (Komet Medical, Lemgo, Germany), is a newly introduced reciprocating motion with software capable of detecting file stress. It changes rotation direction from counterclockwise (CCW) to the reverse direction (CW) if file resistance is excessive [9].

While irrigation may impact cyclic fatigue resistance [10], most laboratory studies are conducted without irrigants or under conditions that do not mimic clinical scenarios such as immersing files in irrigant solutions before testing [11]. Simultaneous irrigation during cyclic fatigue testing seems to provide a more reliable simulation of clinical conditions [10, 12].

Few studies have investigated hybrid motions and limited information is available regarding ReFlex Dynamic reciprocating motion. Moreover, no scientific studies have been published on the effects of ReFlex Dynamic reciprocating motion under continuous irrigation on the cyclic fatigue resistance of nickel-titanium rotary instruments at body temperature. Therefore, this is the first study aimed to evaluate the cyclic fatigue resistance of a single-file system (i.e., Hyflex EDM OneFile) in continuous and ReFlex Dynamic motion with and without irrigation. The null hypotheses were as follows: (1) the kinematics does not affect the cyclic fatigue resistance of Hyflex EDM one file, and (2) continuous irrigation does not affect the cyclic fatigue resistance of the tested files.

## Materials and methods

### Study design

A total of 48 new Hyflex EDM One files (HEDM; #25/variable taper) were used for fatigue resistance tests. The sample size was calculated based on preliminary data using G*Power 3.1.9.2 software (Heinrich Heine-University at Dusseldorf, Dusseldorf, Germany) to achieve 80% power and an alpha error probability of 0.05 [13].

Before testing, all instruments were examined at × 25 magnification under a stereomicroscope (Optika szr 10; Optika Srl, Ponteranica, BG, Italia) to ensure the absence of defects [14, 15]. None of them was excluded. The files were then randomly assigned to four groups (*n* = 12) based on the tested motion and irrigation: continuous rotation and ReFlex Dynamic motion (ReFlex Dynamic^®^, Komet, Breasseler GmbH & Co., Lemgo, Germany), with and without irrigation.

### Experimental cyclic fatigue model

**Fig. 1:**
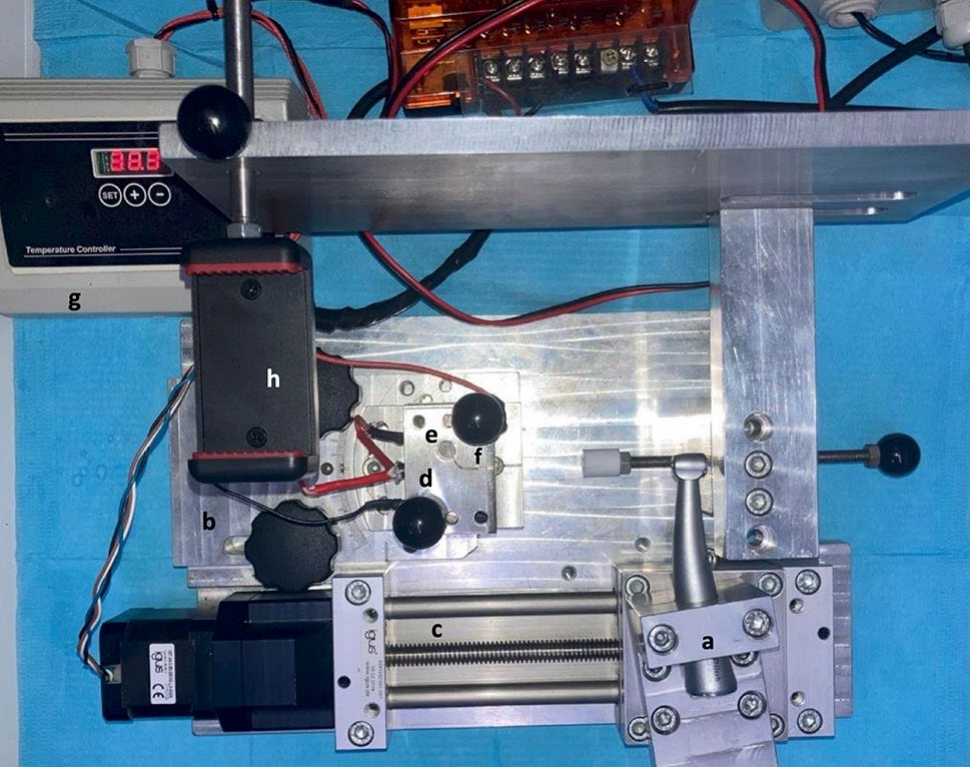
The general setup of the custom-made apparatus for cyclic fatigue tests. **a** The fixed block in which the electric handpiece is inserted; **b** the mobile support on rails, allowing insertion and withdrawal of the file; **c** the support for continuous axial (forward and backward) movement during dynamic testing; **d** the replaceable artificial canal covered by two superimposed Plexiglas slides (for this study, a zirconium canal measuring 16 mm in length, with a 60-degree angle and a curvature radius of 5 mm, was used); **e** the hole in the cover near the canal apex, permitting irrigant outflow during the test through an aspiration system; **f** the insertion point of the irrigant tip; **g** the thermostat, which enabled temperature adjustment via a thermocouple applied to the artificial canal; and **h** the camera support

A custom-made device with a stationary component to secure a 6:1 electric reduction handpiece (EndoPilot^®^ endodontic handpiece) in a stable, fixed three-dimensional position was used for cyclic fatigue tests (Fig. 1) [10, 16]. The setup included a movable rail mount for precise file placement within an artificial canal made of zirconium, measuring 16 mm in length, with a 60-degree angle and a curvature radius of 5 mm [16]. To simulate clinical conditions, a constant temperature of 37 ± 1 °C was maintained by a thermostat connected to the custom machine [17]. In the dynamic cyclic fatigue resistance test, the instruments were tested with a continuous dynamic axial (forward and backward) movement of 3 mm. The ascending and descending movements were set at different speeds to mimic clinical conditions, where the ascending movement is faster for the reduction of resistance. The descending and ascending speeds were 100 and 200 mm/min, respectively [18].

### Cyclic fatigue test

Hyflex EDM One files in continuous rotation were tested at 400 rpm (rotations per minute) and a torque of 2.5 Ncm following the manufacturer’s instructions until failure. Files in ‘ReFlex Dynamic’ were tested following the preset program in the Endopilot motor (Endopilot, Schlumbohm, Brokstedt, Germany). The tests were conducted with and without continuous irrigation, using sodium hypochlorite at room temperature (20 °C) with a concentration of 5.25%. Irrigation flow was set at 0.26 mL/s provided by a 30-G needle and ensured by a hydraulic pump (Tacmina Q series, Awajimachi, Chuo-ku, Osaka, Japan). The irrigation solution’s flow rate was consistently monitored and kept stable throughout all experimental procedures. An aspiration system was turned on during the test to prevent the irrigant from remaining in the system beyond the designated time intervals. For tests without irrigation, a synthetic oil was used as a lubricant in the artificial canal to minimize friction [10]. The time to fracture recorded with a digital chronometer was multiplied by the number of rotations per minute (number of rotations per second) to obtain the number of cycles to fracture (NCF). The length of the fractured fragment was measured using a digital caliper (Digimatic, Mitutoyo Co, Kawasaki, Japan).

### SEM analysis

A field-emission scanning electron microscope (FEG-SEM: Nova NanoSEM 450, FEI Company-Oxford Instruments, Eindhoven, NL) at 400 × and 1200–1600 × magnification was used for SEM analysis to explore failure mechanisms [19].

### Statistical analysis

The normality of data was verified using the Shapiro–Wilk test. Due to the non-normal distribution of the data, they were analyzed using the non-parametric Mann–Whitney test, with the significance level set at 5% (*p* < 0.05). Data analysis was performed using STATA/BE v.17 statistical package (StataCorp LT, College Station, TX, USA).

## Results

The means (standard deviations) as well as the median values [range] of NCF are presented in Table 1.

**Table 1:** Number of cycles to fracture (NCF) of each kinematics tested, with and without irrigation as the mean ± standard deviation and median values [range]

	Number of cycles to fracture (NCF)			
Kinematics	With irrigation		Without Irrigation	
	Mean (SD)	Median [range]	Mean (SD)	Median [range]
Continuous rotation	1157 (98.68)	1153^a1^ [1140,1220]	1214.4 (151.57)	1266^a1^ [1220, 1313]
ReFlex Dynamic motion	775 (33.16)	790^a2^ [750,800]	608 (29.49)	605^b2^ [595,610]

Different superscript letters in the same line show statistically significant differences between instruments activated with the same kinematics with and without irrigation (p < 0.05). Different superscript numbers in the same column indicate statistically significant differences between instruments activated with different kinematics under the same irrigation conditions (p < 0.05)

The tested files in continuous rotation, irrespective of irrigation conditions, exhibited significantly higher NCF compared to those in ReFlex Dynamic (*p* < 0.05).

Irrigation significantly improved the cyclic fatigue resistance of Hyflex EDM OneFile in ReFlex Dynamic (*p* < 0.05), while it had no significant effect on the files tested in continuous rotation (*p* > 0.05).

The length of the fractured file fragments was not statistically different among the tested instruments (mean = 5 ± 0.1 mm) (*p* > 0.05).

**Fig. 2:**
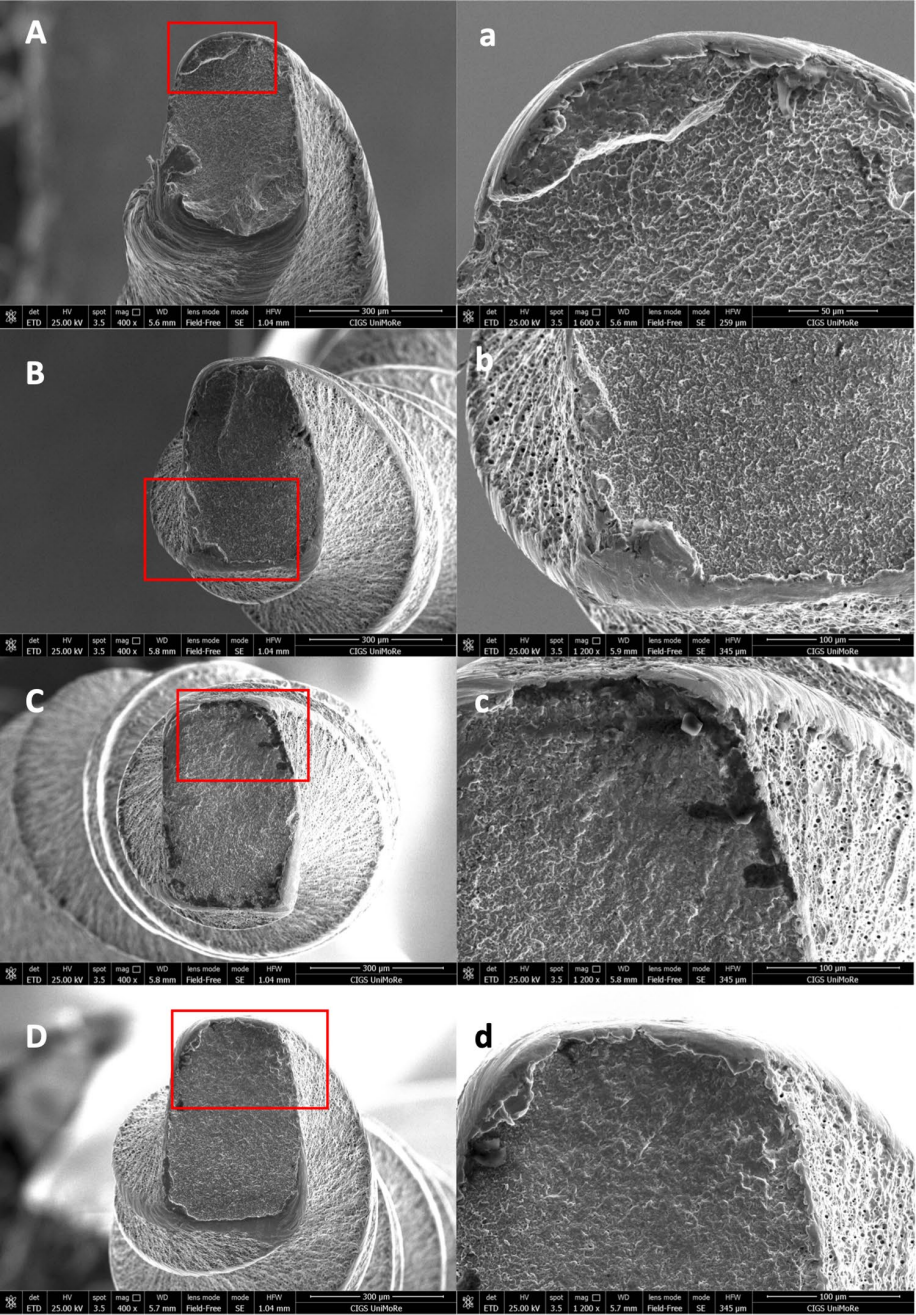
Scanning electron micrographs of the fractured surfaces of HyFlex EDM One files in the axial view (400 × magnification), after cyclic fatigue testing. The crack origins are identified by red rectangular boxes (1200–1600 × magnification). The images reveal numerous dimples scattered across the fractured surfaces, a characteristic feature of ductile fracture. (A, a = Continuous rotation with irrigation; B, b = continuous rotation without irrigation; C, c = ReFlex Dynamic motion with irrigation; D, d = ReFlex Dynamic motion without irrigation)

The SEM micrographs of the fracture surface of the separated fragments revealed typical cyclic fatigue features for all instruments tested (Fig. 2A–D). The distinctive characteristics of a fast fracture zone resulting from excessive stress were noted on the opposite side of the area where the crack originated, aligning with the cyclic fatigue failure mechanism (Fig. 2a–d).

## Discussion

The objective of this study was to assess the cyclic fatigue resistance of a single-file system, specifically the Hyflex EDM OneFile, under a new kinematics (i.e., ReFlex Dynamic motion) and compared it with continuous rotation, with and without simultaneous irrigation.

In recent years, the performance of NiTi instruments has undergone significant modifications to enhance their safety and efficiency in clinical use. The mechanical NiTi files experiment cyclic fatigue when rotating within a curved space, with repetitive mechanical cycles leading to structural changes and eventual file separation [4]. Factors such as file geometry directly influence an instrument’s resistance to cyclic fatigue and torsional stress [20, 21]. To eliminate these variables, the same NiTi files were used and the temperature was set to simulate clinical conditions at 37 °C [4]. Specifically, HEDM files were selected for their widespread clinical use, high cyclic fatigue resistance, and flexibility as heat-treated instruments [22]. Although the novel motion was originally designed for a different type of instrument, it was applied here to evaluate its performance with flexible, heat-treated files, providing valuable insights into its clinical application.

A customized machine ensured the reproducibility of the tests, using a zirconium artificial canal to avoid galvanic phenomena under NaOCl irrigation [10]. The correct instrument placement in the artificial canal was confirmed by the similar length of fractured segments.

Natural extracted teeth were not used due to difficulties in achieving standardization and ensuring suitable clinical conditions [4]. Furthermore, it is notable that only dynamic devices can replicate the pecking motion carried out by the operator [9]. The dynamic test, involving axial movements, closely replicates real clinical conditions. Axial motion, mimicking a pecking motion, prolongs the instrument’s lifespan by distributing stress, unlike the static cyclic fatigue resistance test that concentrates stress in a single area [18].

ReFlex uses an advanced motion system that transitions from rotary to reciprocating motion upon detecting resistance, reducing torsional stress on the file. It operates in two modes: ReFlex Smart, which continuously adapts to torque resistance to minimize file stress, making it suitable for complex canals, and ReFlex Dynamic, which operates at a higher rotation speed until torque resistance is detected, at which point it switches to a reciprocating motion [23]. The ReFlex Dynamic motion was selected due to its innovative mechanics and the lack of studies comparing it with continuous rotation. While only three studies [9, 14, 24] in literature deal with ReFlex motion and cyclic fatigue, they are not directly comparable with our results due to the different files and kinematics.

The NCF values of instruments in continuous rotation were significantly higher than those observed with ReFlex Dynamic motion; thus, the first null hypothesis can be rejected. This difference could be attributed to the fact that, in reciprocating motion, the CW and CCW angles are specifically tailored to endodontic reciprocating systems. To prevent exceeding elastic deformation, the CCW angle must remain smaller than the elastic limit of the system material [9]. Supporting this, the study by Zubizarreta-Macho et al. (2021) compared ‘Smart’ and ‘Dynamic’ ReFlex motions with a reciprocal alternative movement [9]. Their findings revealed that the ReFlex Dynamic motion was more effective than ReFlex Smart and reciprocation when stiffer instruments were used. However, with heat-treated instruments or those designed to enhance flexibility, the kinematics showed less effective performance [9, 25]. These observations align with the study by Generali et al. [25], which demonstrated that the time to fracture increased for instruments operating with ReFlex Dynamic motion when non-heat-treated and stiffer instruments were used. Despite these findings, further research is necessary to validate these interpretations.

Irrigation with NaOCl significantly increased the NCF values of the files tested in ReFlex Dynamic motion; for this reason, the second hypothesis is only partially rejected. This effect may be explained by the continuous irrigation method employed in our study, which likely provided a lubricating effect, reducing friction and heat generation. These findings differ from those of Huang et al. [26], who reported no statistically significant differences among instruments tested with 5.25% NaOCl and water. The discrepancy may be attributed to methodological differences, as Huang et al. used a pre-immersion approach, whereas our study applied continuous irrigation during the test. This effect was observed only for the ReFlex Dynamic motion, probably because instruments under this motion experience greater flexural stress compared to continuous rotation, making them more sensitive to the lubricating effect of NaOCl.

The scanning electron microscopic examination revealed a characteristic fractographic pattern consistent with cyclic fatigue fracture, with comparable visual results across the groups evaluated. The images highlighted crack initiation zones, while the fractured surfaces displayed multiple dimples.

The study holds clinical significance as it was carried out in rigorous laboratory conditions designed to mimic in vivo scenarios. This methodology enables the extrapolation of valuable insights into how the cyclic fatigue resistance of flexible instruments is influenced by the novel hybrid kinematic and the environmental conditions. However, considering the limitations of in vitro studies, further research considering anatomical root variability and operator variables is needed. Validation of these in vitro findings and additional examinations with various irrigants and instruments are recommended. A comparison with other ReFlex motions (i.e., Reflex Smart) could also be explored.

## Conclusions

Under the limitations of this in vitro study, HyFlex EDM One files under continuous rotation demonstrated higher resistance to cyclic fatigue compared to those in ReFlex Dynamic, regardless of irrigation. Continuous hypochlorite irrigation significantly improved only the cyclic fatigue resistance of the same files tested in ReFlex Dynamic motion.

## Data Availability

Data are available from the corresponding author upon request.
